# Prevalence and incidence of venous thromboembolism in geriatric patients admitted to long-term care hospitals

**DOI:** 10.1038/s41598-024-67480-1

**Published:** 2024-07-31

**Authors:** Gernot Wagner, Daniel Steiner, Gerald Ohrenberger, Michael Smeikal, Christoph Gisinger, Deddo Moertl, Stephan Nopp, Gerald Gartlehner, Ingrid Pabinger, Cihan Ay

**Affiliations:** 1 Department for Evidence-Based Medicine and Evaluation, University for Continuing Education Krems, Krems, Austria; 2 Clinical Division of Haematology and Haemostaseology, Department of Medicine I, Medical University of Vienna, Vienna, Austria; 3 Haus der Barmherzigkeit Seeboeckgasse, Vienna, Austria; 4 Haus der Barmherzigkeit Tokiostraße, Vienna, Austria; 5 Center for Geriatric Medicine and Geriatric Nursing, University for Continuing Education Krems, Krems, Austria; 6 Department of Internal Medicine 3, University Hospital St. Poelten, St. Poelten, Austria; 7 Karl Landsteiner University of Health Sciences, Krems, Austria; 8 Center for Public Health Methods, RTI International, Research Triangle Park, NC USA; 9 Department of Obstetrics, Gynecology and Perinatal Medicine, I. M., Sechenov First Moscow State Medical University (Sechenov University), Moscow, Russia

**Keywords:** Venous thromboembolism, Oral anticoagulation, Long-term care hospital, Geriatric patients, Medical research, Epidemiology

## Abstract

The risk of venous thromboembolism (VTE) increases with age. However, the risk of VTE in the setting of long-term care hospitals is understudied. Our objective was to provide data on the prevalence and incidence of VTE in older adults admitted to long-term care hospitals. In this retrospective cohort study, we collected data about chronically ill and multimorbid patients aged 65 years and older from two long-term care hospitals. The primary endpoint of this study was the lifetime prevalence of VTE, and the secondary endpoint was VTE incidence during residency in long-term care hospitals. We analysed data from 1148 patients with a mean age of 84.1 ± 7.9 years, of whom 74.2% were women. The lifetime prevalence of VTE at baseline was 9.6% (95% CI 7.9–11.4). Cumulative incidence of VTE at 1, 2, and 3 years from baseline was estimated at 3.5% (95% CI 2.5–4.7), 4.2% (95% CI 3.1–5.5), and 5.4% (95% CI 4.1–7.0), respectively. Overall, the incidence rate of VTE in our study was 2.82 (95% CI 2.18–3.66) per 100 person-years. The study indicated a considerably high lifetime prevalence and incidence of VTE during residence in long-term care hospital settings, requiring further evaluation in larger prospective studies.

## Introduction

Venous thromboembolism (VTE), which includes pulmonary embolism (PE) and deep vein thrombosis (DVT), is the third most prevalent cardiovascular disease, with an incidence rate of approximately 0.1–0.2 per 100 person-years in the general population^1–3^. The occurrence of VTE is associated with a significant risk of complications, such as increased risk of mortality, bleeding due to anticoagulation, recurrent VTE, post-thrombotic syndrome, impaired physical function, and reduced quality of life^3–8^. Consequently, VTE poses a considerable disease burden, impacting individuals, society, and the economy^2,9–12^.

Commonly recognised risk factors for developing VTE include persistent factors, such as active cancer; major transient factors, such as surgery; and minor transient factors, such as admission to hospital for fewer than three days^13^. The incidence of VTE rises exponentially with age, placing older and frail persons at increased risk^2,14^. Many of these older persons live in nursing homes or are admitted to long-term care hospitals since the ability to care for themselves is limited. Compared to nursing homes, long-term care hospitals provide continuous medical care by physicians, including regular ward rounds. In addition to advanced age, a high proportion of these geriatric patients suffer from various comorbidities, such as chronic kidney and respiratory diseases, adding further to the high baseline VTE risk^15^. Therefore, long-term care hospital patients may have VTE risk levels at least as high as those in medical inpatients. Due to the sociodemographic changes in our ageing society, a steady and considerable increase in the number of nursing home and long-term care hospital residents is expected^16^.

Previous studies have shown a substantial incidence of VTE in nursing home residents, up to 3.65 events per 100 person-years^17,18^. However, the VTE risk of this vulnerable patient population, particularly in the long-term care hospital setting, is still understudied. Therefore, the aim of this study was to provide data on rates of VTE in older adults admitted to long-term care hospitals.

## Methods

### Study design

We performed a cohort study and retrospectively analysed routinely collected data from two long-term care hospitals in Vienna, Austria. The Ethics Committee of the Medical University of Vienna approved this study (EK Nr. 2124/2017) and waived the need for informed consent for this retrospective data analysis. We conducted this study in accordance with the Declaration of Helsinki and the Good Clinical Practice (GCP) guidelines. In terms of reporting, we followed the Strengthening the Reporting of Observational Studies in Epidemiology (STROBE) statement^19^.

### Patient population

We collected data from chronically ill and multimorbid patients who were admitted to two long-term care hospitals, namely Haus der Barmherzigkeit Tokiostrasse and Haus der Barmherzigkeit Seeboeckgasse in Vienna, Austria. The data collection period ranged from 1 January 2014 to 31 October 2017. For our cohort study, patients 65 years or older with varying levels of care dependency and underlying chronic medical conditions were eligible. Usually, patients admitted to a long-term care hospital are those with multimorbidity (e.g., COPD, heart failure, frailty, stroke, diabetes) requiring continuous medical care at a level that cannot be provided in a nursing home. We retrieved data from electronic medical records about comorbidities, geriatric assessment, medication, laboratory results, date of admission, discharge, and death. The Charlson Comorbidity Index^20^ was calculated for each patient based on available comorbidity data. For inpatients on 1 January 2014, comorbidities and endpoints were considered as past medical history if dated on or before this date. For patients admitted between 1 January 2014 and 31 October 31 2017, diagnoses were considered as past medical history if dated prior to admission or up to five days after admission. We identified prescribed oral anticoagulants (non-vitamin K oral anticoagulants [NOAC], vitamin K antagonist [VKA]), parenteral anticoagulants (low-molecular-weight-heparin [LMWH], fondaparinux), and platelet inhibitors according to the Anatomical Therapeutic Chemical classification system. Medication was considered present at baseline if it was prescribed up to five days after 1 January 2014 or after admission. The findings on the prevalence and incidence of atrial fibrillation in this cohort have been previously published^21^.

### Endpoints

The primary endpoint of this study was the lifetime prevalence of VTE at baseline, and the secondary endpoint was the incidence of VTE during residence in long-term care hospitals. VTE was defined as any provoked (i.e., presence of any obvious cause or risk factor) or unprovoked (i.e., absence of any obvious cause or risk factor) pelvic or lower extremity DVT and/or PE. Using International Classification of Disease, 10^th^ Revision (ICD-10) codes assigned by treating physicians and/or free-text information from electronic medical records, we identified patients who experienced pelvic or lower extremity DVT (ICD-10 codes I80.1, I80.2, I80.3) and/or PE (ICD-10 code I26.-). When necessary, two clinicians involved in this study manually reviewed medical records to clarify the diagnoses and dates of outcome events.

### Statistical analysis

Comprehensive descriptive statistics for baseline and endpoint variables were performed. For continuous data, we computed means and standard deviations if approximately normally distributed; otherwise, the median and the 25th and 75th percentiles were calculated. Categorical data were summarised as absolute and relative frequencies. We determined the lifetime prevalence of VTE at baseline (i.e., 1 January 2014 or date of admission for those hospitalised in the period from 2 January 2014 and 31 October 31 2017) with a 95% confidence interval (CI) by calculating the proportion of patients with history of VTE during their lifetime.

Regarding the incidence of VTE, we estimated the cumulative incidence and considered death from any cause as the competing risk event^22^. We calculated the time from baseline to occurrence of VTE (date of diagnosis or first documentation in the electronic medical record). If no VTE was observed, we calculated the time until discharge from a long-term care hospital, death, or the end of the follow-up period (31 October 2017). We determined incidence rates by dividing the number of VTE events by years at risk in person-time, considering the observation period from baseline to discharge, death, or the end of the follow-up period (October 31, 2017) as the person-time at risk. We performed stratified analyses for incidence rates according to history of VTE (lifetime history versus no history of VTE) and the presence of anticoagulation (oral anticoagulation versus parenteral anticoagulation versus no anticoagulation) at baseline. Statistical analysis for this study was conducted using SAS® software version 9.4 (SAS Institute Inc., Cary, NC, USA) and Stata Statistical Software: Release 17.0 (StataCorp LLC, College Station, TX, USA).

## Results

**Figure 1:**
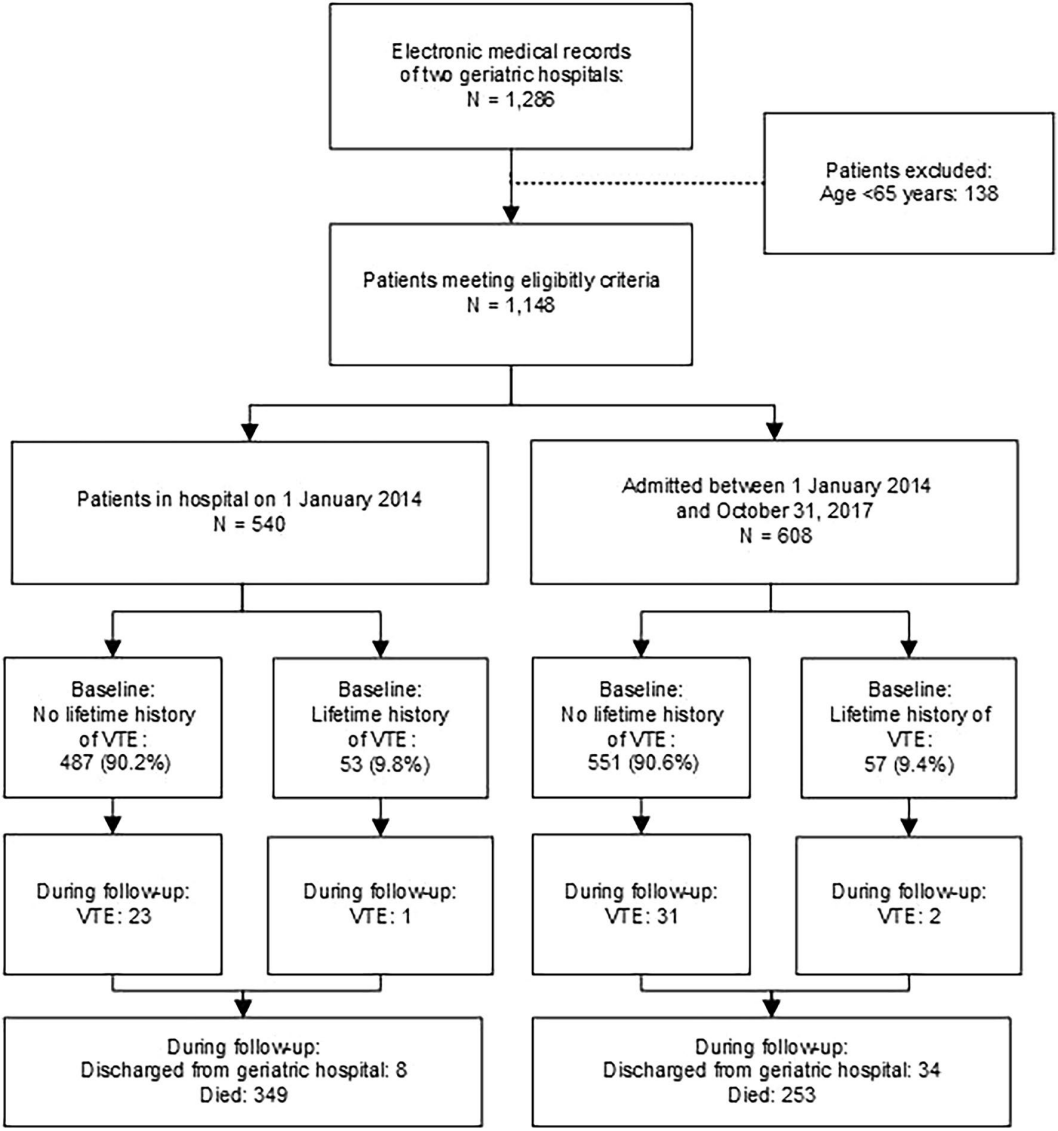
Study flow chart showing the number of patients with VTE at baseline and during follow-up. VTE, venous thromboembolism; N, number of patients.

A total of 1,148 patients from two long-term care hospitals were included in the analysis. Figure 1 shows the number of patients with and without VTE at baseline and during the follow-up period. In total, 42 out of 1,148 patients (3.7%) were discharged from long-term care hospitals or transferred to another institution, and 602 patients (52.4%) died during the study period.

### Patient characteristics

The mean age of the patients was 84.1 ± 7.9 years; 74.2% of the patients were female. More than half of the patients (51.0%) were 85 years or older, and more than 80% of patients had a Charlson Comorbidity Index below 4. Among the patients with a history of VTE, 32.7% (36 out of 110) received oral anticoagulants, and 15.5% (17 out of 110) received parenteral anticoagulants. Table 1 shows the baseline characteristics of patients with and without a history of at least one VTE. Supplementary Table S1 presents the patients’ baseline characteristics by sex.

**Table 1:** Patients’ baseline characteristics overall and according to lifetime history of VTE.

	All	No lifetime history of VTE	Lifetime history of VTE
	N = 1,148	N = 1,038	N = 110
Age, years, mean ± SD	84.1 ± 7.9	84.2 ± 7.9	84.0 ± 8.1
65–74	189 (16.5)	169 (16.3)	20 (18.2)
75–84	373 (32.5)	337 (32.5)	36 (32.7)
≥ 85	586 (51.1)	532 (51.3)	54 (49.1)
Female, N (%)	852 (74.2)	761 (73.3)	91 (82.7)
BMI, kg/m^2^, mean ± SD (*105 missing*)	25.0 ± 5.7	24.9 ± 5.7	25.7 ± 5.2
Charlson Comorbidity Index, N (%)			
0–1	443 (38.6)	405 (39.0)	38 (34.6)
2–3	512 (44.6)	460 (44.3)	52 (47.3)
≥ 4	193 (16.8)	173 (16.7)	20 (18.2)
High care dependency^a^, N (%) (*50 missing*)	725 (66.0)	654 (65.9)	71 (67.0)
Medical history, N (%)			
Hypertension	777 (67.7)	702 (67.6)	75 (68.2)
Hyperlipidemia	243 (21.2)	226 (21.8)	17 (15.5)
Diabetes mellitus	312 (27.2)	277 (26.7)	35 (31.8)
Chronic renal insufficiency	266 (23.2)	238 (22.9)	28 (25.5)
Ischaemic heart disease	288 (25.1)	253 (24.4)	35 (31.8)
Heart failure/Cardiomyopathy	202 (17.6)	175 (16.9)	27 (24.6)
Atrial fibrillation	347 (30.2)	317 (30.5)	30 (27.3)
Previous stroke or TIA	316 (27.5)	288 (27.8)	28 (25.5)
Peripheral artery disease	91 (7.9)	83 (8.0)	8 (7.3)
Solid or haematologic malignancy^b^	167 (14.6)	144 (13.9)	23 (20.9)
Bleeding^c^	126 (11.0)	110 (10.6)	16 (14.6)
Dementia	649 (56.5)	588 (56.7)	61 (55.5)
Antithrombotic therapy, n (%)			
VKA	61 (5.3)	54 (5.2)	7 (6.4)
NOAC	134 (11.7)	105 (10.1)	29 (26.4)
Rivaroxaban	100 (8.7)	78 (7.5)	22 (20.0)
10 mg	5 (0.4)	5 (0.5)	0
15 mg	58 (5.1)	47 (4.5)	11 (10.0)
20 mg	37 (3.2)	26 (2.5)	11 (10.0)
Dabigatran	13 (1.1)	10 (1.0)	3 (2.7)
75 mg	1 (0.1)	1 (0.1)	0
110 mg	9 (0.8)	7 (0.7)	2 (1.8)
150 mg	3 (0.3)	2 (0.2)	1 (0.9)
Apixaban	14 (1.2)	10 (1.0)	4 (3.6)
2.5 mg	10 (0.9)	6 (0.6)	4 (3.6)
5.0 mg	4 (0.4)	4 (0.4)	0
Edoxaban	7 (0.6)	7 (0.7)	0
30 mg	4 (0.4)	4 (0.4)	0
60 mg	3 (0.3)	3 (0.3)	0
LMWH	181 (15.8)	164 (15.8)	17 (15.5)
Fondaparinux	2 (0.2)	2 (0.2)	0
ASS	247 (21.5)	230 (22.2)	17 (15.5)
Clopidogrel	70 (6.1)	65 (6.3)	5 (4.6)
Prasugrel or Ticagrelor	2 (0.2)	1 (0.1)	1 (0.9)

*AF*, atrial fibrillation; *ASS*, acetylsalicylic acid; *BMI*, body mass index; *IQR*, interquartile range; *LMWH*, low-molecular-weight-heparin; *SD*, standard deviation; *N*, number of patients, *NOAC*, non-vitamin K antagonist oral anticoagulant; *TIA*, transient ischaemic attack; *VKA*, vitamin K antagonists; *VTE*, venous thromboembolism.

^a^Based on assessment with the care dependency scale (CDS).

^b^Excluding non-melanoma skin cancer. Patients with multiple malignancies were counted only once.

^c^Bleeding was defined as any overt traumatic or nontraumatic intracranial, gastrointestinal, or other extracranial bleeding (e.g., ocular, skin and soft tissue, renal, retroperitoneal, pericardial, intra-articular).

### Prevalence of venous thromboembolism

At baseline, of 1,148 patients, 110 had a history of VTE during their lifetime: 60 had experienced DVT, 34 PE, and 16 both DVT and PE. Consequently, the baseline lifetime prevalence of VTE was determined to be 9.6% (95% CI 7.9–11.4).

### Incidence of venous thromboembolism

Over a median follow-up of 3.7 years, we observed newly diagnosed VTE in 57 patients (5.0%). Among them, 47 patients developed DVT, 8 PE, and 2 both DVT and PE. Competing risk analysis revealed cumulative 1-, 2-, and 3-year incidences of venous thromboembolism of 3.5% (95% CI 2.5–4.7), 4.2% (95% CI 3.1–5.5), and 5.4% (95% CI 4.1–7.0), respectively. When considering only patients without a history of VTE in the competing risk analysis (N = 1,038), the cumulative 1-, 2-, and 3-year incidences of VTE were 3.7% (95% CI 2.6–5.0), 4.4% (95% CI 3.2–5.8), and 5.6% (95% CI 4.2–7.3), respectively.

The incidence rate of VTE in the entire study population was 2.82 (95% CI 2.18–3.66) per 100 person-years. Among patients without a history of VTE, the incidence rate was 2.94 (95% CI 2.25–3.84) per 100 person-years. During the follow-up period, among the 110 patients with a history of VTE at baseline, 3 patients experienced recurrent VTE.

In analyses stratified according to treatment regimens at baseline, the incidence rate of VTE per 100 person-years was found to be 1.09 (95% CI 0.41–2.91) in patients receiving oral anticoagulation, 2.34 (95% CI 0.98–5.63) in patients treated with LMWH, and 3.34 (95% CI 2.51–4.43) in patients without anticoagulant therapy.

Results of a subgroup analysis stratified by sex are provided in the supplementary material (Tables S2-S4).

### Treatment patterns of VTE

Table 2 presents the antithrombotic regimens in patients who experienced VTE during the follow-up period. Most of the patients received LMWH, followed by NOAC.

**Table 2:** Antithrombotic therapy in patients with newly diagnosed VTE during follow-up.

Antithrombotic therapy	Patients with new VTE N (%)^a^
	N = 57
None	1 (1.8)^b^
VKA	2 (3.5)
Apixaban	6 (10.5)
Dabigatran	0
Edoxaban	1 (1.8)
Rivaroxaban	14 (24.6)
LMWH	33 (57.9)

Abbreviations: NOAC, non-vitamin K antagonist oral anticoagulant; LMWH, low-molecular-weight-heparin; N, number of patients; VKA, vitamin K antagonists; VTE, venous thromboembolism.

^a^Patients who initially received LMWH or fondaparinux and subsequently switched to oral anticoagulation were considered in the category of prescribed oral anticoagulation.

^b^One patient died from pulmonary embolism, so no long-term anticoagulation therapy was recorded.

## Discussion

In this retrospective cohort study of geriatric patients admitted to long-term care hospitals, we identified a high lifetime prevalence of VTE of approximately 10%, along with an overall incidence rate of 2.82 per 100 person-years during their stay in long-term care hospitals.

Comparing our findings to previous studies conducted on nursing home residents, we observed a higher prevalence of VTE. For instance, a study from the US reported a history of VTE diagnosis on admission in 3.7% of all admissions across 181 nursing homes^23^. It should be noted that the patients included in this study were younger and had fewer comorbidities compared to our study population. In addition, comparability of our study is limited due to the methodological differences, e.g., the aforementioned study included patients from nursing homes (compared to long-term care hospitals in our study), and the outcome was based on Minimum Data Set (MDS) 2.0 assessments^23^.

The incidence of VTE observed in our study is not only significantly higher compared to the general population^1,2^, but it also exceeds population-wide estimates for individuals above the age of 80, which range around 0.8 VTE events per 100 person-years^14^. However, when considering studies performed in a nursing home setting, our findings demonstrate comparable incidence rates, indicating a substantial burden of VTE in geriatric patient populations.

While we were unable to find specific data from long-term care hospitals, there are previous studies that assessed VTE incidence using administrative data from one or more nursing homes and reported incidence rates ranging from 1.3 per 100 person-years^24^ up to 3.7 per 100 person-years^23^. An observational cohort study conducted in the UK found rates between 0.71 and 2.48 per 100 person-years, depending on the inclusion of probable and possible VTE events^17^. Similarly, the authors of a cohort study of nursing home residents in Israel observed incidence rates between 1.4 and 1.6 per 100 person-years^25^. The most recently published study, including nursing home residents in the US, revealed an incidence rate of 3.65 per 100 person-years^18^. Proposed reasons for the variation in VTE incidence across these studies include differences in patient characteristics, setting, follow-up duration, and the availability of and access to proper diagnostic methods^23^. The higher incidence of VTE in our study compared to the general population and its similarity to rates reported in nursing home studies indicate a substantial burden of VTE in older adults admitted to long-term care hospitals.

The high incidence of VTE observed in our study can be attributed to several factors. The geriatric population in long-term care hospitals is characterised by a high prevalence of chronic illnesses, including malignancies, which are known to increase the risk of VTE. This underlying comorbidity burden contributes significantly to the elevated incidence of VTE in this specific population. Moreover, the setting of long-term care hospitals differs in several important aspects from other healthcare institutions, such as nursing homes. Notably, long-term care hospitals provide continuous medical care by physicians, including regular ward rounds. This increased level of medical attention and close monitoring may enhance the detection rate of clinical events, including VTE, compared to other healthcare settings.

It is important to note that the immediate and accurate diagnosis of VTE has a significant impact on epidemiological findings. While a correct diagnosis is typically made promptly in symptomatic patients, the possibility of overlooking VTE in the absence of typical symptoms exists. Consequently, the incidence of VTE might have been underestimated in our study due to the presence of clinically silent thrombotic events. This hypothesis is supported by the findings of an asymptomatic VTE point prevalence of 18% in chronically ill patients who underwent systematic screening^26^. Moreover, an autopsy study revealed that PE was the cause of death in 8% of residents in a chronic care institution^27^, highlighting the high rate of missed diagnoses prior to death. These data emphasise the crucial role of VTE detection in the context of long-term care settings and highlight the necessity for increased attention regarding its correct diagnosis.

The use of prophylactic or therapeutic anticoagulation could have potentially reduced the risk of thrombosis in individual patients in our study. However, the overall frequency of antithrombotic therapy in patients with a history of VTE at baseline was relatively low. This could be explained by several factors, including the fact that some of the VTE events may have occurred long ago, leading to the discontinuation of therapy, or the VTE events were provoked by transient risk factors and treatment was performed for a limited duration. However, patients in our study also had other indications for anticoagulation, such as atrial fibrillation^21^. Finally, the variable follow-up duration among the patients in our study could have potentially affected the incidence rates observed.

Our observed decrease in VTE incidence rates among patients stratified by oral anticoagulant therapy, parenteral anticoagulation, and no anticoagulation should be interpreted with caution. Due to the small sample size and the presence of confounding factors, it does not provide reliable evidence to draw any conclusions regarding VTE prevention. The subgroup of patients receiving oral anticoagulation included individuals on therapeutic anticoagulation for reasons unrelated to VTE, such as atrial fibrillation, and the parenteral anticoagulation subgroup comprised both prophylactic and therapeutic uses. In addition, clinical decisions regarding anticoagulation and relevant information such as clinical presentation, patient preference, estimated life expectancy, and bleeding risk were not available from our data set. Finally, we assessed the use of anticoagulation at baseline and at the time point of a new VTE diagnosis during follow-up. We were not able to take into account changes in anticoagulation during follow-up as a time-dependent co-variate.

In contrast to our findings, other studies have reported significantly lower rates of anticoagulant usage, ranging from 3^25^ to 6%^17^. However, it is important to note that these studies had different designs and did not specifically evaluate the incidence rates of VTE in relation to anticoagulant use. For example, the study by Gatt et al. was a historical cohort study conducted in a nursing home which did not specify the time point of medication assessment^25^. Apenteng et al. included more than 1000 nursing home patients in a prospective cohort study, in which approximately 6% of patients received anticoagulation at baseline, primarily for atrial fibrillation^17^.

While there are valuable data regarding thromboprophylaxis in hospitalised medical and surgical patients, the available evidence for long-term care residents and patients admitted to long-term care hospitals is insufficient. Furthermore, to our knowledge, no clinical prediction tool is available to aid the decision-making process. Therefore, the most appropriate approach at present is to engage in individualised, patient-centred discussions when considering thromboprophylaxis. These discussions should take into account the cumulative impact of risk factors for VTE while also carefully assessing the risk of bleeding^28,29^. Additionally, patients’ values and preferences should be acknowledged in shared decision making.

Our study has several limitations that should be considered. First, it is susceptible to bias due to its retrospective design with data from electronic medical records. Second, the generalizability of our findings from two long-term care hospitals to other healthcare settings, such as nursing homes or acute care hospitals, is limited. The specific characteristics and patient populations in long-term care hospitals differ from those in other healthcare facilities. Third, our analysis regarding the usage of anticoagulants and occurrence of new VTE during follow-up is exploratory in nature. Insufficient information regarding dosage, indication, and duration of anticoagulation limits our ability to draw any conclusions. We were not able to evaluate the underlying reason for the decisions for or against anticoagulation, as this information was not available in the data set. Finally, we could not obtain information on whether the VTE events were provoked or unprovoked.

In conclusion, our study highlights the significant burden of VTE among older adults admitted to long-term care hospitals. To confirm our findings on the prevalence and incidence of VTE in long-term care hospitals, there is a need for large prospective studies. Future studies might advance our understanding of VTE in this vulnerable patient population, support physicians in making more informed decisions, and optimise the prevention of VTE in long-term care settings.

### Supplementary Information

Supplementary Tables.

## Data Availability

The data set analysed during the current study is available from the corresponding author on reasonable request.
