# Supplementary material for: Prevalence and incidence of venous thromboembolism in geriatric patients admitted to long-term care hospitals
Source: Sci Rep. 2024 Jul 31;14:17737. doi: 10.1038/s41598-024-67480-1 (PMC11291751; doi:10.1038/s41598-024-67480-1)
Supplement: Supplementary file 1 — Supplementary Tables. [file 41598_2024_67480_MOESM1_ESM.docx]

**Prevalence and incidence of venous thromboembolism in geriatric patients admitted to long-term care hospitals**

Gernot Wagner, Daniel Steiner, Gerald Ohrenberger, Michael Smeikal, Christoph Gisinger, Deddo Moertl, Stephan Nopp, Gerald Gartlehner, Ingrid Pabinger, and Cihan Ay

# Supplementary Material

**Table S1.** Patients’ baseline characteristics according to lifetime history of VTE and sex

|  | **No lifetime history**  **of VTE**  **N = 1,038** | | **Lifetime history**  **of VTE**  **N = 110** | |
| --- | --- | --- | --- | --- |
|  | **Male** | **Female** | **Male** | **Female** |
|  | **N =277**  **(26.7%)** | **N = 761**  **(73.3%)** | **N =19**  **(17.3%)** | **N =91**  **(82.7%)** |
| Age, years, mean ± SD | 81.1 ± 7.7 | 85.3 ± 7.7 | 79.7 ± 7.7 | 84.9 ± 8.0 |
| 65–74 | 70 (25.3) | 99 (13.0) | 6 (31.6) | 14 (15.4) |
| 75–84 | 106 (38.3) | 231 (30.4) | 8 (42.1) | 28 (30.8) |
| ≥85 | 101 (36.5) | 431 (56.6) | 5 (26.3) | 49 (53.9) |
| BMI, kg/m^2^, mean ± SD (*105 missing*) | 25.4 ± 5.3 | 24.8 ± 5.9 | 24.7 ± 5.0 | 25.9 ± 5.3 |
| Charlson Comorbidity Index, N (%) |  |  |  |  |
| 0–1 | 98 (35.4) | 307 (40.3) | 4 (21.1) | 34 (37.4) |
| 2–3 | 116 (41.9) | 344 (45.2) | 11 (57.9) | 41 (45.1) |
| ≥4 | 63 (22.7) | 110 (14.5) | 4 (21.1) | 16 (17.6) |
| High care dependency^a^, N (%) (*50 missing*) | 181 (67.8) | 473 (65.2) | 16 (88.9) | 55 (62.5) |
| **Medical history, N (%)** |  |  |  |  |
| Hypertension | 178 (64.3) | 524 (68.9) | 12 (63.2) | 63 (69.2) |
| Hyperlipidemia | 58 (20.9) | 168 (22.1) | 2 (10.5) | 15 (16.5) |
| Diabetes mellitus | 84 (30.3) | 193 (25.4) | 8 (42.1) | 27 (29.7) |
| Chronic renal insufficiency | 57 (20.6) | 181 (23.8) | 5 (26.3) | 23 (25.3) |
| Ischaemic heart disease | 78 (28.2) | 175 (23.0) | 6 (31.6) | 29 (31.9) |
| Heart failure / Cardiomyopathy | 36 (13.0) | 139 (18.3) | 3 (15.8) | 24 (26.4) |
| Atrial fibrillation | 87 (31.4) | 230 (30.2) | 5 (26.3) | 25 (27.5) |
| Previous stroke or TIA | 93 (33.6) | 195 (25.6) | 9 (47.4) | 19 (20.9) |
| Peripheral artery disease | 36 (13.0) | 47 (6.2) | 2 (10.5) | 6 (6.6) |
| Solid or haematologic malignancy^b^ | 41 (14.8) | 103 (13.5) | 4 (21.1) | 19 (20.9) |
| Bleeding^c^ | 36 (13.0) | 74 (9.7) | 5 (26.3) | 11 (12.1) |
| Dementia | 161 (58.1) | 427 (56.1) | 7 (36.8) | 54 (59.3) |
| **Antithrombotic therapy, n (%)** |  |  |  |  |
| VKA | 12 (4.3) | 42 (5.5) | 2 (10.5) | 5 (5.5) |
| NOAC | 31 (11.2) | 74 (9.7) | 4 (21.1) | 25 (27.5) |
| LMWH | 43 (15.5) | 121 (15.9) | 2 (10.5) | 15 (16.5) |
| Fondaparinux | 0 | 2 (0.3) | 0 | 0 |
| ASS | 66 (23.8) | 164 (21.6) | 4 (21.1) | 13 (14.3) |
| Clopidogrel | 21 (7.6) | 44 (5.8) | 1 (5.3) | 4 (4.4) |
| Prasugrel or Ticagrelor | 0 | 1 (0.1) |  | 0 |

Abbreviations: AF, atrial fibrillation; ASS, acetylsalicylic acid; BMI, body mass index; IQR, interquartile range; LMWH, low-molecular-weight-heparin; SD, standard deviation; N, number of patients, NOAC, non-vitamin K antagonist oral anticoagulant; TIA, transient ischaemic attack; VKA, vitamin K antagonists; VTE, venous thromboembolism

^a^ Based on assessment with the care dependency scale (CDS)

^b^ Excluding non-melanoma skin cancer. Patients with multiple malignancies were counted only once.

^c^ Bleeding was defined as any clinically overt traumatic or nontraumatic intracranial, gastrointestinal, or other extracranial bleeding (e.g., ocular, skin and soft tissue, renal, retroperitoneal, pericardial, intra-articular).

**Table S2.** Lifetime prevalence of VTE at baseline overall and by sex

| **Characteristic** | **No. of patients with lifetime history of VTE at baseline** | **Lifetime prevalence of VTE**  **at baseline (95% CI)** |
| --- | --- | --- |
| All (N = 1148) | 110 | 9.6% (7.9–11.4) |
| Female (N = 852) | 91 | 10.7% (8.7-13.0) |
| Male (N = 296) | 19 | 6.4% (3.9-9.8) |

Abbreviations: CI, confidence interval; VTE, venous thromboembolism; No., number; N, number of patients

**Table S3.** Number of events and cumulative 1-, 2-, and 3-year incidences of VTE by sex

| **Characteristic** | **No. of patients with newly diagnosed VTE during follow-up** | **Cumulative incidence**  **of VTE (95% CI)** |
| --- | --- | --- |
| All (N = 1148) |  |  |
| 1 Year | 38 | 3.5% (2.5–4.7) |
| 2 Years | 44 | 4.2% (3.1–5.5) |
| 3 Years | 54 | 5.4% (4.1–7.0) |
| Female (N = 852) |  |  |
| 1 Year | 29 | 3.6% (2.5-5.0) |
| 2 Years | 34 | 4.3% (3.0-5.9) |
| 3 Years | 43 | 5.8% (4.3-7.7) |
| Male (N = 296) |  |  |
| 1 Year | 9 | 3.2% (1.6-5.8) |
| 2 Years | 10 | 3.7% (1.9-6.5) |
| 3 Years | 11 | 4.3% (2.2-7.3) |

Abbreviations: CI, confidence interval; VTE, venous thromboembolism; No., number; N, number of patients

**Table S4.** Incidence rate of VTE per 100 person-years overall and by sex

| **Characteristic** | **No. of patients with newly diagnosed VTE during follow-up** | **Person-years** | **Incidence rate per 100 person-years (95% CI)** |
| --- | --- | --- | --- |
| All (N = 1148) | 57 | 2018 | 2.82 (2.18-3.66) |
| Female (N = 852) | 46 | 1562 | 2.94 (2.21-3.93) |
| Male (N = 296) | 11 | 456 | 2.41 (1.34-4.36) |

Abbreviations: CI, confidence interval; VTE, venous thromboembolism; No., number; N, number of patients
